# Inhibitory Effect of Ursolic Acid on Ultraviolet B Radiation-Induced Oxidative Stress and Proinflammatory Response-Mediated Senescence in Human Skin Dermal Fibroblasts

**DOI:** 10.1155/2020/1246510

**Published:** 2020-06-15

**Authors:** Ramachandran Samivel, Rajendra Prasad Nagarajan, Umadevi Subramanian, Adnan Ali Khan, Ali Masmali, Turki Almubrad, Saeed Akhtar

**Affiliations:** ^1^Cornea Research Chair, Department of Optometry, College of Applied Medical Sciences, King Saud University, Saudi Arabia; ^2^Department of Biochemistry and Biotechnology, Faculty of Science, Annamalai University, Tamil Nadu, India; ^3^Translational Research Platform for Veterinary Biologicals, Central University Laboratory Building, TANUVAS, Tamil Nadu, India

## Abstract

Ultraviolet radiation is an environmental carcinogenic agent that enhances inflammation and immunological reactions in the exposed human skin cells leading to oxidative photoaging of the epidermal and dermal segment. In the present study, we investigated the protective role of ursolic acid (UA) against ultraviolet B (UVB) radiation- induced photoaging an *in vitro* model of human skin dermal fibroblasts. UA-pretreated human skin dermal fibroblast (HDF) cells were exposed to UVB radiation to evaluated cell viability, reactive oxygen species (ROS), mitochondrial membrane potential, lipid peroxidation, antioxidant status, DNA damage, proinflammatory response, apoptotic induction, and matrix metalloproteinase (MMP) alteration. The UA pretreatment of HDFs mitigated the UVB irradiation-induced cytotoxicity, ROS generation, and mitochondrial membrane potential alteration and lipid peroxidation, depletion of antioxidant status, DNA damage, and apoptotic induction. UA pretreatment of HDFs also attenuated the UVB-induced expression of inflammatory (TNF-*α* and NF-*κ*B) and apoptotic (p53, Bax, and caspase-3) and MMPs (MMP-2 and MMP-9) and enhanced the Bcl-2 protein levels in 20 *μ*M UA treatment, when compared to concentrations. Hence, these results revealed that UA has the potential to mitigate UVB-induced extracellular damage by interfering with the ROS-mediated apoptotic induction and photoaging senescence and thus is a potential therapeutic agent to protect the skin against UVB-irradiation induced photooxidative damage.

## 1. Introduction

Ultraviolet radiation, predominantly UVB (290–320 nm wavelength), can be hazardous to human health by inducing cyclobutane pyrimidine dimer (CPD) formation, cellular damage, inflammation, and tissue photodamage [1]. The UVB radiation causes massive production of photooxidative damage by reactive oxygen species (ROS), including hydroxyl radicals, superoxide anions, singlet oxygen, and hydrogen peroxide anion-exposed cells [2]. The increased ROS load can mitigate intracellular organelles to cause oxidative damage to cellular proteins, lipids, and DNA. This leads to photooxidative damage and the activation of apoptotic signal transduction stimulus that can promote photoaging and skin carcinogenesis. Higher concentrations of ROS generated by UVA and UVB irradiation, through the direct absorption of UVB photons by DNA, lead to structural changes in the skin. During the generation of ROS from UV irradiation, photons are absorbed by endogenous photosensitizer molecules, which react with oxygen resulting in increases of various free radicals in the epidermal and dermal portion causing damage to the skin cells [3].

The human skin contains predominantly collagen, elastin, proteoglycans, and fibronectin [4]. Collagen fibrils and elastin are structural integrity proteins responsible for the strength and elasticity of the skin, and their disarrangement due to photodamage causes aging of the skin [5]. Fibroblasts are cells of dermal origin that provide durability and flexibility to the skin and connective tissues. The skin contains many mechanisms for protection against oxidative damage, such as antioxidants, catalase (CAT), superoxide dismutase (SOD), glutathione peroxidase (GPx), and reduced glutathione (GSH) [6], that protect the cells from UVB-induced oxidative damage. However, when the ROS concentration overcomes these antioxidant defenses, cell oxidation damage occurs [7]. UVB irradiation-induced oxidative damage appears to play a major role in a number of specific pathological conditions where the dermal matrix is degraded [8].

UVB irradiation leads to changes in the elastic-collagenous tissues of the skin by breaking down the core collagen fibrils, a major component of the extracellular matrix [9, 10]. These alterations in the extracellular matrix are possibly mediated by MMPs that are known to cause premature wrinkling and aged skin [11]. UVB irradiation is the primary agent known to generate the reactive oxygen species (ROS) triggering the DNA damage [12]. It has been shown that the ROS increases MMP expression to alleviate early senescence in human dermal fibroblasts [13].

However, additional efforts are needed to prevent serious pathological consequences of UVB exposure. In particular, daily intake of dietary antioxidants or treatment of the skin with natural products containing antioxidant ingredients may be a useful strategy for the prevention of UVB irradiation-mediated skin damage [14]. Furthermore, since artificial sunscreen agents may generate free radicals themselves when activated by UV light and only few of them can provide full spectral protection against ultraviolet radiation, the use of formulations containing plant-derived antioxidants may have highly beneficial effects for the prevention of skin photoaging. Higher plants are naturally exposed to high doses of solar UV radiation; thus, they have developed a number of defense mechanisms against UV-induced damage, such as the capability to absorb UV radiation by accumulation of phenolic compounds in their superficial layers [15]. Several plant phytochemicals have gained considerable attention as skin photoprotective agents [16]. In particular, *triterpenoids* represent an interesting class of active polyphenolic phytochemicals that protect the UV light-induced skin damage due to their wide spectrum of actions including anti-inflammatory, antioxidant, antimutagenic, and anticarcinogenic properties as well as modulation of enzyme activities [17].

**Figure 1:**
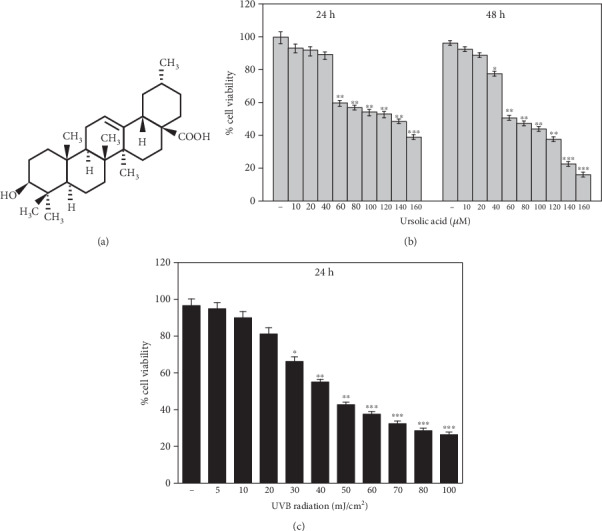
(a) Structure of ursolic acid (UA); (b) stereological evaluation of the viability of pretreatment of HDF cells with UA at concentrations from 0 to 160 *μ*M for 24- and 48-hour incubation. (c) Stereological evaluation of the viability of HDF cells treated with UVB. The cell viability was not affected by irradiation dose < 40 mJ/cm^2^ whereas it gradually decreased by the exposure of UVB irradiation >40 mJ/cm^2^ for 24 hours. Values are given as mean ± SD of three experiments in each group; statistical analysis was performed by one-way ANOVA followed by DMRT at *P* < 0.05, *P* < 0.01, and *P* < 0.001 compared to the nonirradiated control. *P* values not sharing a common marking (∗, ∗∗, and ∗∗∗) differ significantly (DMRT).

Ursolic acid (UA; Figure 1(a)) is a lipophilic, most abundant pentacyclic triterpenoid, widely present in natural medicinal plants, such as *Rosmarinus officinalis*, *Ocimum basilicum*, *Mentha spicata*, and *Veronica serpyllifolia*. It is also present in fruits, such as *Pyrus malus*, *Vaccinium oxycoccos*, and *Prunus domestica*, some edible fruits, and food ingredients [18, 19]. UA has been shown to exhibit diverse biological activities such as antioxidant, anticarcinogenic, anti-inflammatory, antifibrotic, renoprotective, hepatoprotective, cardioprotective, chemopreventive, immunomodulatory, antibacterial, antihyperlipidemic, hypoglycemic, and antimutagenic activities [20–22]. In the present study, we investigated the protective effect of UA against UVB irradiation-induced oxidative stress-mediated activation of inflammatory response and apoptotic signaling cascades triggering the photoaging in HDFs *in vitro*.

## 2. Materials and Methods

### 2.1. Chemicals

Ursolic acid (purity ≥ 98%), thiobarbituric acid (TBA), phenazinemethosulphate (PMS), nitroblue tetrazolium (NBT), 5,5-dithiobis 2-nitrobenzoic acid (DTNB), 3-(4, 5-dimethyl-2-thiaozolyl)-2,5-diphenyl-2H tetrazolium bromide (MTT), 2′7′-diacetyl dichlorofluorescein (DCFH-DA), rhodamine-123, and nicotinamide adenine dinucleotide (NAD) were supplied by Sigma-Aldrich, St. Louis, USA. The mouse monoclonal antibodies anti-TNF-*α*, anti-NF-*κ*B (p65), anti-p53, anti-Bax, anti-Bcl-2, anti-caspase-3, anti-MMP-2, anti-MMP-9, anti-*β*-actin, and goat anti-mouse IgG-HRP polyclonal antibody were also purchased from Sigma-Aldrich, St. Louis, USA. RNA isolation kit, cDNA synthesis kit, PCR primers, and PCR master mix were purchased from Qiagen, USA. Bovine serum albumin (BSA), RIPA buffer, low melting agarose (LMPA), normal melting agarose (NMPA), phosphate buffered saline (PBS) and reduced glutathione (GSH) were purchased from Merck, India. All other chemicals, gradient solvents, and other analytical grades were acquired from S.D Fine Chemical and Himedia, India.

### 2.2. Human Skin Dermal Fibroblast Cell Culture

HDF cells were purchased from Invitrogen Bioservices (Catalogue No: C0135C), India. MEM/F12 medium, Low Serum Growth Supplement (LSGS; Cat No: S-003-10) kit, trypsin/EDTA solution, and trypsin neutralizer solution were obtained from Casecade Biologics, Invitrogen cell culture, India. The primary HDF cells were propagated in MEM/F12 medium supplemented with the LSSG kit containing 2% *v*/*v* fetal bovine serum, 1 *μ*g/mL hydrocortisone, 10 ng/mL human epidermal growth factor, 3 ng/mL basic fibroblast growth factor, 10 *μ*g/mL heparin, and 1% penicillin and streptomycin solution. Cell cultures were maintained in a humidified 95% O_2_ and 5% CO_2_ incubator at 37°C for 7-14 days until they reached 85% confluence. Then, the cells were subcultured and used in further experiments.

### 2.3. Determination of Optimum Dosage of Ursolic Acid and UVB Radiation

The 3-(4, 5-dimethyl-2-thiaozolyl)-2,5-diphenyl-2H tetrazolium bromide (MTT) assay is a colorimetric nonradioactive assay for measuring the cell viability by increased metabolization of tetrazolium salt [23]. 24 h fibroblast cultures were treated with different concentrations of UA (10 to 160 *μ*M) and kept incubated for 24 and 48 hours. Different concentrations of UA were added with culture media and incubated for the dose-dependent study. In the control group, DMSO (0.05%) alone was used. Similarly, cells were treated with various doses of UVB irradiation (5-100 mJ/cm^2^) and incubated at 24 hours. The cytotoxicity was measured by MTT assay in both the studies. LD_50_ values were calculated, and the optimum dose of UA and UVB irradiation was used for further studies.

#### 2.3.1. Experimental Protocol

Cultured human dermal fibroblasts were divided into six groups such as Group 1: control (nonirradiated and nontreated fibroblasts); Group 2: nonirradiated fibroblasts treated with 20 *μ*M of UA; Group 3: human skin dermal fibroblasts exposed to 40 mJ/cm^2^ UVB irradiation; Group 4: human skin dermal fibroblasts treated with 10 *μ*M of UA plus 40 mJ/cm^2^ UVB irradiation; Group 5: human skin dermal fibroblasts treated with 20 *μ*M of UA plus 40 mJ/cm^2^ UVB irradiation; and Group 6: human skin dermal fibroblasts treated with 40 *μ*M of UA plus 40 mJ/cm^2^ UVB irradiation.

#### 2.3.2. Treatment of the UA in HDF Cells

Before thirty minutes prior UVB irradiation, HDF cells in the above groups were pretreated with three doses of UA (10, 20, and 40 *μ*M). Initially, the cell viability by trypan blue dye exclusion was carried out to confirm whether UA itself (10, 20, and 40 *μ*M) caused any cell death. Before exposure to UVB light, the cells were washed thrice with PBS. There was no decrease in cell viability in control (non-UVB-irradiated fibroblasts) cells over the experimental periods.

#### 2.3.3. UVB Irradiation Procedure

For UVB irradiation, the cell culture medium was removed from the plates and the cells were washed with PBS thrice to ensure that components of the culture medium would not interfere with the irradiation procedure. The culture plates with cells in a thin layer of PBS were covered with a UV permeable membrane to prevent contamination. A battery of TL 20 W/20 fluorescent tubes served as the UVB source (F8T5 UVB lamp; Japan). The wavelength range was set at 290-320 nm and peaked at 312 nm and with an intensity of 2.2 mW/cm^2^ for 30 min. The exposure rate of UVB irradiation (W/cm^2^ into J/cm^2^) was calculated using the following formula: *T* (min) = *J* (cm^2^) × 16.7/(mW/cm^2^). The output of UVB irradiation dose from the lamp was measured using a radiometer. The total UVB irradiation dose was exposed 0.05 to 0.4 J/cm^2^ at different times. Control samples were processed identically but were not UVB irradiated.

### 2.4. MTT Assay

Cultured fibroblasts 1 × 10^6^ cells/mL were seeded into 96-well plates and pretreated with three different concentrations of UA (10, 20, and 40 *μ*M). After 1 h incubation with/or without UA, the cells were exposed to UVB irradiation. Then, the cells were incubated at 37°C, 5% CO_2_ and 95% O_2_, for 24 h. MTT (0.5 mg/mL; 20 *μ*L/well) was added, and the cells were further incubated for 4 h. 200 *μ*L of DMSO was added in each well, and the plates were centrifuged at 1200 rpm for 5 minutes. The optical density of the cell supernatant was measured using a microplate reader at 540 nm.

### 2.5. Quantification of Intracellular Reactive Oxygen Species (ROS) Levels

The intracellular ROS level was quantified by using 2′7′-diacetyl dichlorofluorescein diacetate (DCFH-DA) fluorescence probe for directly measuring the redox state of a cell [24]. Fibroblasts pretreated with UA with or without UVB irradiation were incubated 10 *μ*M/mL DCFH-DA (dissolved in PBS) in 6-well plates for 15 minutes, followed by three washes with PBS. Fluorescence intensity was determined at 488/525 nm by a spectrofluorometer, and the images were captured by an Olympus BX53 fluorescence microscope.

### 2.6. Change in Mitochondrial Transmembrane Potential (*Δψ*m)

Cationic dyes are incorporated electrophoretically into the mitochondrial matrix in response to the electric potential across the inner mitochondrial membrane during oxidative phosphorylation. The alterations of cell membrane potential (*Δψ*m) have been evaluated by measuring rhodamine-123 fluorescence dissipate by a spectrofluorometer and microscopy [25]. Rhodamine-123 was dissolved in ethanol, and 1 *μ*L Rh-123 (10 mmol/L) was added to fibroblasts that were UA pretreated with/or without UVB irradiation in 6-well plates. Cells were incubated for 30 min, washed thrice with PBS, and viewed under a fluorescence microscope using blue filter (450-490 nm). Polarized mitochondria are marked by orange-red fluorescence, and depolarized mitochondria are marked by green fluorescence. The fluorescence intensity was quantified by a spectrofluorometer.

### 2.7. Estimation of Lipid Peroxidation and Cellular Antioxidant Status

HDF cells were scraped off in 1 mL homogenate buffer (50 mM Tris-HCl, 150 mM KCl, 2 mM EDTA, 3 mM DL-dithiothreitol, and 0.2% Triton-X 100) using a cell scraper. The cell suspension was collected and sonicated for 15 sec in 40 cycles. The homogenates were centrifuged at 12,000 g for 30 min at 4°C, and the supernatant was stored in separate aliquots at -80°C until biochemical analysis. The level of membrane lipid peroxidation was determined by analyzing TBA-reactive substance according to the protocol of Niehaus and Samuelsons [26]. The pink-colored chromogen formed by the reaction of 2-TBA with breakdown products of lipid peroxidation was measured.

The total superoxide dismutase (SOD) enzyme activity was quantified by the method of Kakkar et al. [27], based on the inhibition of the NADH-PMS-NBT complex formation. Catalase (CAT) activity was assayed by the method of Sinha [28], by quantifying the hydrogen peroxide levels after reacting with dichromate in acetic acid. The activity of glutathione peroxidase (GPx) was measured by the method of Rotruck et al. [29]. In this assay, a known amount of enzyme preparation was allowed to react with hydrogen peroxide (H_2_O_2_) and GSH for a specified time period. Then, the GSH content after the reaction was measured. The total GSH content was measured by the method of Ellman [30]. This method was based on the development of a yellow color, when 5,5-dithiobis 2-nitrobenzoic acid was added to a compound containing sulfhydryl groups.

### 2.8. Alkaline Single-Cell Gel Electrophoresis (Comet Assay)

DNA damage was estimated by alkaline single-cell gel electrophoresis (comet assay) according to the method of Singh et al. [31]. The experimental protocol was described in our previous research article by Ramachandran et al. [32]. For the analysis of the comet images, the extent of DNA damage was estimated by fluorescence microscopy using the digital camera and analyzed by CASP image analysis software, CASP. For each sample, 100 cells were analyzed, and DNA damage was quantified by % head DNA, % tail DNA, tail moment, tail length, and Olive tail moment.

### 2.9. Detection of Apoptotic Nuclei by AO/EB Staining

Acridine orange/ethidium bromide (AO/EB) dual staining was performed to detect morphological changes upon apoptotic induction in UA-pretreated and UVB-irradiated cells [33]. The cells were fixed in a 3 : 1 ratio of methanol and glacial acetic acid for 1 h at room temperature. The cells were labeled with a 1 : 1 ratio of AO (100 *μ*g/mL) and EB (100 *μ*g/mL) in PBS and incubated for 5 min; then, the excess unbinding dye was removed by washing with PBS. Stained cells were visualized under UV illumination using the 40x objective (BX53 Olympus fluorescence microscope), and the digitized images were captured. The apoptotic cells, with the shrunken, nuclear fragmentation, brightly fluorescent, apoptotic nuclei, were easily detected through their high fluorescence and condensed chromatin; ethidium bromide-positive nuclei were scored, and the percentage of apoptotic cells was calculated.

### 2.10. Reverse Transcription-Polymerase Chain Reaction

Total cellular RNA was extracted using Trizol reagent according to the manufacturer's instructions (RNeasy Mini Kit, Qiagen). One microgram of total RNA from each sample was annealed for 5 min at 70°C with 500 ng oligo (dT) and reverse transcribed to cDNA per 20 *μ*L reaction for 1 h at 42°C. The reaction was stopped by heating for 10 min at 70°C. The PCR was performed on the resultant cDNA from each sample with specific primers for human MMP-2, MMP-9, and glyceraldehyde-3-phosphate dehydrogenase (GAPDH), which severed as an internal control. PCR amplification was performed with a thermocycler (Applied Biosystems, ABI). The 25 *μ*L reaction mixtures consist of 2 *μ*L cDNA, 1 *μ*L forward and reverse primer, and 12.5 *μ*L 2x PCR master mix. The annealing temperature and cycle for both MMP-2 and MMP-9 were 55°C and 40 cycles. At the end of amplification, the reaction mixture was heated for 10 minutes at 72°C and then cooled to 4°C. A 10 *μ*L sample of each PCR product was separated by performing gel electrophoresis on 2% agarose containing ethidium bromide. The 2% agarose gel was analyzed under ultraviolet light against the DNA molecular length markers; the signal was detected using the Alpha Innotech imaging system (San Lorenzo, CA).

### 2.11. Western Blot Analysis for Proinflammatory and Apoptotic Marker Expression

Western blotting assay was performed as previously described [34]. Briefly, the cell lysates were prepared from HDFs in 1x Laemmli lysis buffer (2.4 M glycerol, 0.14 M Tris-HCl (pH 6.8), 0.21 M sodium dodecyl sulfate, and 0.3 mM bromophenol blue) and boiled for 5 minutes. The protein content was measured using BCA protein assay reagent (Pierce, Rockford, IL, USA). Protein samples (30 *μ*g/well) were diluted with 1x lysis buffer, separated by SDS-PAGE, and transferred onto PVDF membranes (semidry transfer unit, Bio-Rad). The primary antibodies including TNF-*α* (1 : 1000), NF-*κ*B (p65; 1 : 1000), p53 (1 : 1000), Bax (1 : 1000), Bcl-2 (1 : 1000), caspase-3 (1 : 1000), MMP-2 (1 : 1000), MMP-9 (1 : 1000), and *β*-actin (1: 2000) were added to the membranes and incubated at 4°C overnight. The membranes were washed three times for 5 minutes in Tris-buffered solution with Tween20 (TBST) and incubated with HRP-conjugated secondary antibody (1 : 5000) for 1 h at room temperature. Finally, membranes were washed three times for 5 minutes in TBST and ECL solution was added to develop the signals. The images were acquired by Image Station 2000R (Kodak, NY, USA).

### 2.12. Statistical Analysis

Statistical analysis was done using SPSS (Statistical Package for Social Sciences) version 16 software. The values were presented as mean ± standard deviation (M ± SD), and data were analyzed using one-way analysis of variance (ANOVA) and Duncan's Multiple Range Test (DMRT) to correlate the difference among the variables. All the values were expressed as means of six (*n* = 6) for determination; the significance was established at ^∗^*P* < 0.05, ^∗∗^*P* < 0.01, and ^∗∗∗^*P* < 0.001 levels compared to the control group.

## 3. Results

### 3.1. Effect of UA and UVB in HDF Cells

The percentage cytotoxicity of HDFs gradually increased by the pretreatment of the UA with concentrations from 60 to 160 *μ*M at 24 and 48 h. Cell viability was not affected by the UA concentrations from 0 to 40 *μ*M (Figure 1(b)). The UVB-irradiated doses of 5-100 mJ/cm^2^ on the cultured HDFs for 24 h also increased the cytotoxicity of the cells. The irradiation dose range < 40 mJ/cm^2^ significantly reduced the cell viability by more than 50% (Figure 1(c)).

**Figure 2:**
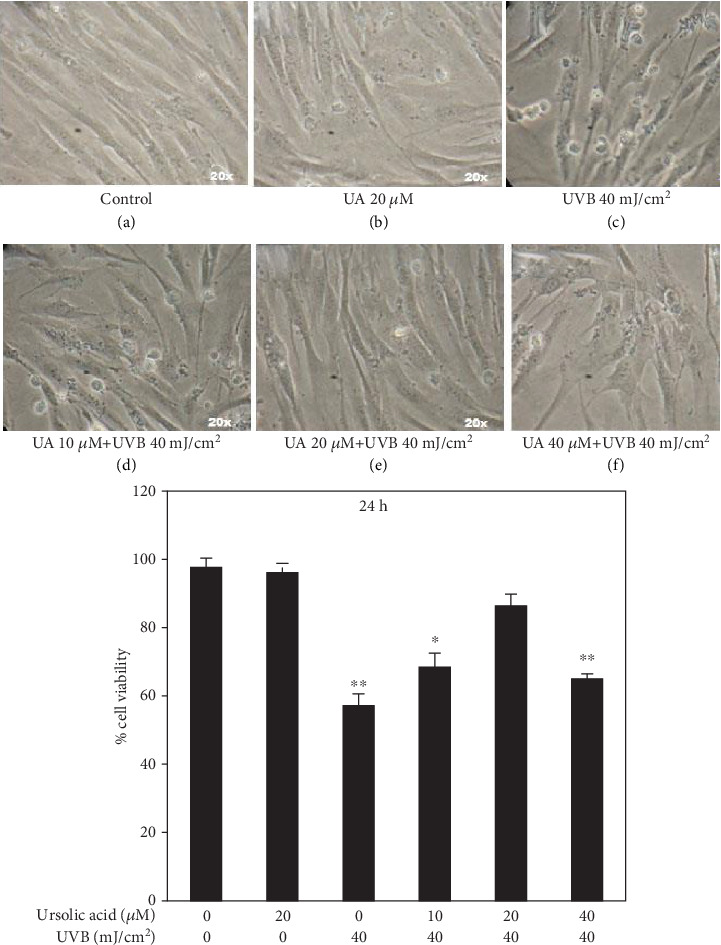
Stereological evaluation of the viability of HDFs treated with UA, UVB-irradiated, and UA+UVB-irradiated cells. Values are given as mean ± SD of three experiments in each group; statistical analysis was performed by one-way ANOVA followed by DMRT at *P* < 0.05, *P* < 0.01, and *P* < 0.001 compared to the nonirradiated control. *P* values not sharing a common marking (∗, ∗∗, and ∗∗∗) differ significantly (DMRT).

Viability was not affected for control untreated cells and cells treated with 20 *μ*M UA (Figures 2(a) and 2(b)), whereas the viability of HDFs was reduced at UVB 40 mJ/cm^2^ irradiation (Figure 2(c)). The HDFs' viability was significantly (*P* = 0.01) increased in the “UA and UVB irradiation” (UA 10 *μ*M+UVB 40 mJ/cm^2^ and UA 20 *μ*M+UVB 40 mJ/cm^2^) groups compared to UVB-irradiated HDFs that did not receive UA pretreatment (Figures 2(d)–2(f)).

### 3.2. UA Prevents Intracellular ROS Generation in HDFs

**Figure 3:**
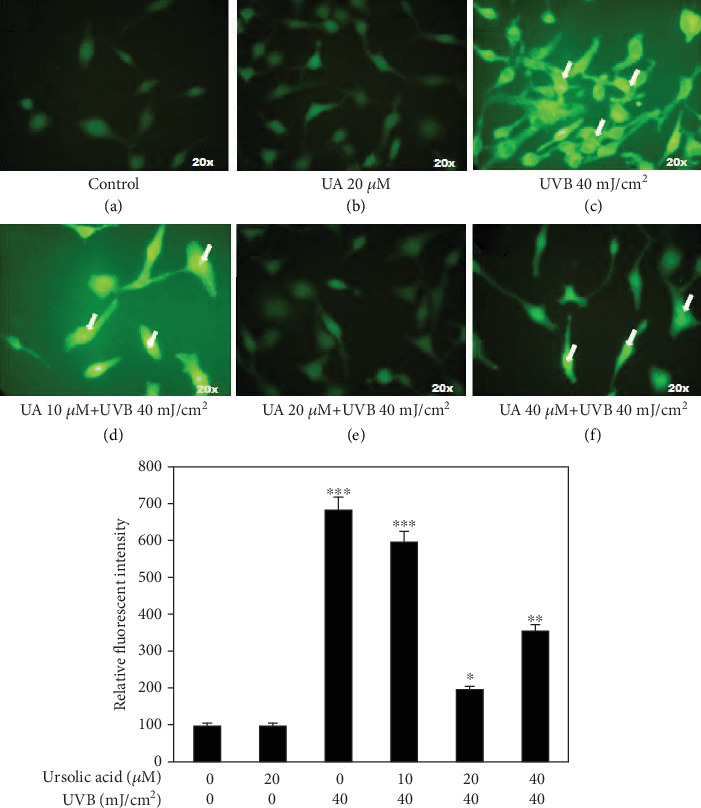
Fluorescent microscopy imaging in DCFH staining of HDF cells treated with UA, UVB irradiation, and UA+UVB irradiation to assess intracellular ROS generation: (a, b) no fluorescent DCFH stain indicating that ROS generation was not affected by UA pretreatment at 20 *μ*M concentration; (c, d) strong DCFH fluorescent staining indicating that ROS generation of HDFs was increased at UVB 40 mJ/cm^2^ irradiation and UA 10 *μ*M+UVB 40 mJ/cm^2^; (e, f) low DCFH staining indicating that the ROS generation was decreased by mixture of UA and UVB irradiation (UA 20 *μ*M+UVB 40 mJ/cm^2^ and UA 40 *μ*M+UVB 40 mJ/Cm^2^). All images were captured at 20x magnification. Values are given as mean ± SD of three experiments in each group; statistical analysis was performed by one-way ANOVA followed by DMRT at *P* < 0.05, *P* < 0.01, and *P* < 0.001 compared to the nonirradiated control. *P* values not sharing a common marking (∗, ∗∗, and ∗∗∗) differ significantly (DMRT).

Fluorescence microscopic digital image projected lack of staining in the control group and cells treated with UA 20 *μ*M (Figures 3(a) and 3(b)). The HDFs exposed to “UVB radiation” had distinguished dense staining indicative of a markedly increased level of intracellular ROS generation at the single-cell level (Figure 3(c)). Thus, UVB radiation-mediated induction of ROS was most likely responsible for ≥50% cell death of HDFs at 40 mJ/cm^2^. When the HDFs were pretreated with 20 *μ*M UA prior to the UVB radiation (“UA+UVB irradiation”), they showed the absence of DCFDA staining, indicating reduced generation of ROS level (*P* = 0.01) in HDF cells (Figures 3(e) and 3(f)) compared to that in the UVB-irradiated group. These results suggest that this polyphenolic compound is capable of scavenging intracellular ROS in fibroblasts that were generated due to UVB irradiation-induced oxidative stress.

### 3.3. UA Prevents UVB-Induced Mitochondrial Membrane Potential (*Δψ*m) Alteration in HDFs

**Figure 4:**
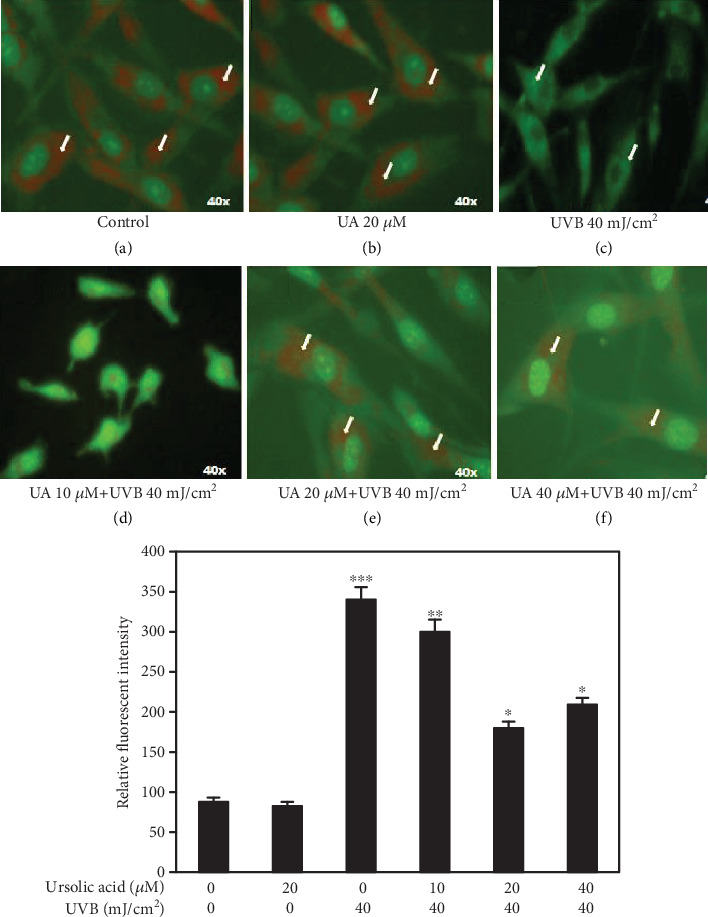
Fluorescent microscopy imaging in rhodamine-123 staining of HDF cells treated with UA, UVB radiation, and UA+UVB radiation to assess the mitochondrial membrane potential (*Δψ*m); (a, b) lack of green staining indicating that *Δψ*m in HDFs was not affected by UA 20 *μ*M and in control cells; (c, d) gradual green staining of Rh-123 suggesting *Δψ*m was severely affected by the treatment of UVB 40 mJ/cm^2^ and UA 10 *μ*M+UVB 40 mJ/cm^2^; (e, f) lack of green staining indicating that *Δψ*m increased by the treatment of UA 20 *μ*M+UVB 40 mJ/cm^2^ and UA 40 *μ*M+UVB 40 mJ/cm^2^. All images were captured at 40x magnification. Values are given as mean ± SD of three experiments in each group; statistical analysis was performed by one-way ANOVA followed by DMRT at *P* < 0.05, *P* < 0.01, and *P* < 0.001 compared to the nonirradiated control. *P* values not sharing a common marking (∗, ∗∗, and ∗∗∗) differ significantly (DMRT).

Mitochondrial membrane potential changes were observed under a fluorescence microscope by Rh-123 staining. The images showed the accumulation of the red Rh-123 dye in control and UA 20 *μ*M treated cells, indicating an increased level of polarization in HDF cells (Figures 4(a) and 4(b)). UVB-irradiated (UVB 40 mJ/Cm^2^) HDFs emitted green fluorescence suggesting an increased depolarization of *Δψ*m (Figure 4(c)). The pretreatment of UA (20 and 40 *μ*M) prior to the UVB irradiation of the HDF cells enhanced membrane polarization significantly (*P* = 0.01) as showed by red fluorescence (Figures 4(e) and 4(f)) compared to that of the UVB irradiation group.

### 3.4. Biochemical Changes of UA-Treated HDF Cells

**Figure 5:**
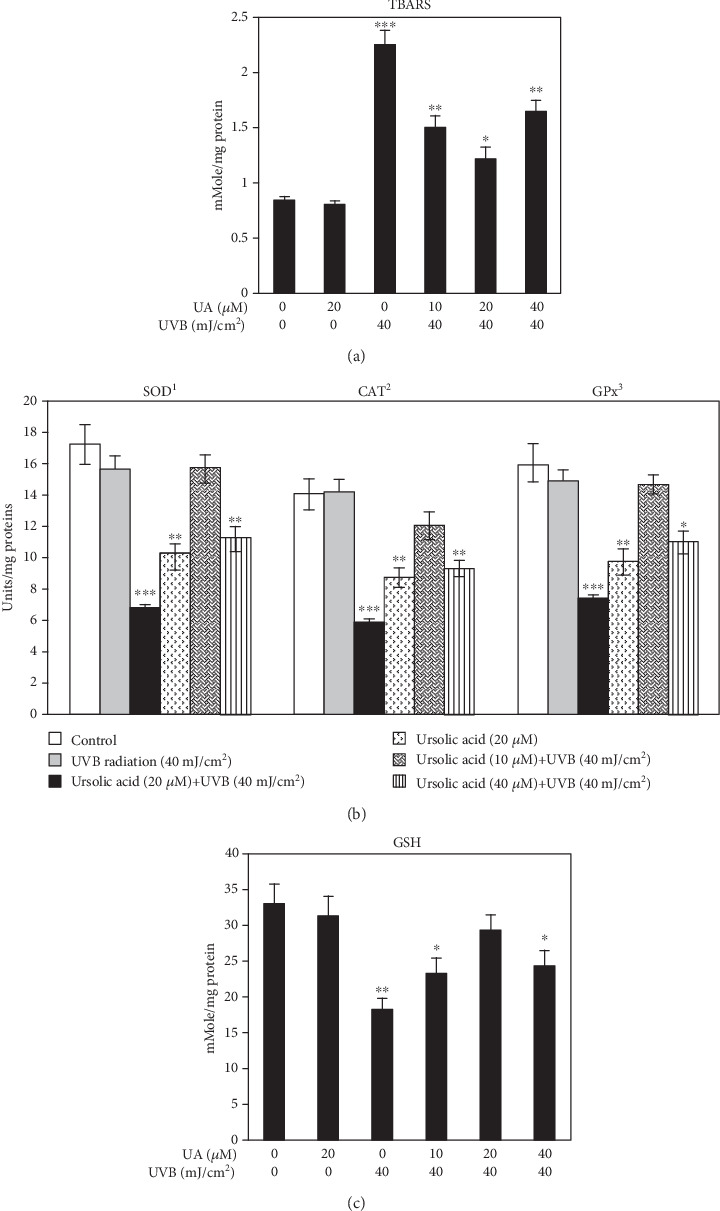
(a) Evaluation of TBARS (lipid peroxidation) in HDFs treated with UA, UVB radiation, and UA+UVB radiation. (b) Assessment of intracellular SOD, CAT, and GPx activities of HDF cells treated with UA, UVB radiation, and UA+UVB radiation. ^1^Enzyme concentration required for 50% inhibition of nitroblue tetrazolium reduction in one minute. ^2^*μ*mol of hydrogen peroxide consumed per minute. ^3^*μ*g of glutathione consumed per minute. (c) Evaluation of endogenous GSH level in HDFs treated with UA, UVB radiation, and UA+UVB radiation. Bars represent mean ± SD of six separate experiments (*n* = 6 per group). Statistical analysis was performed by one-way ANOVA followed by DMRT at *P* < 0.05, *P* < 0.01, and *P* < 0.001 compared to the nonirradiated control. *P* values not sharing a common marking (∗, ∗∗, and ∗∗∗) differ significantly (DMRT).

The results of the biochemical analysis of lipid peroxidation and antioxidant parameters are shown in Figures 5(a)–5(c). TBARS (lipid peroxidation) was not affected in control and UA 20 *μ*M treated groups, whereas its level was significantly increased in UVB 40 mJ/cm^2^ irradiated HDF cells (Figure 5(a)) compared to that in control cells. The pretreatment of HDFs with UA (10, 20, and 40 *μ*M) prior to the exposure to UVB 40 mJ/cm^2^ radiation significantly decreased the levels of TBARS (compared with the control group, *P* < 0.05). Among the three different doses tested, 20 *μ*M of UA showed maximum protection against UVB-induced oxidative lipid peroxidation in HDFs (Figure 5(a)).

The enzymatic antioxidant activities of SOD, CAT, and GPx (Figure 5(b)) and nonenzymatic antioxidant GSH (Figure 5(c)) levels were decreased in HDFs treated with 40 mJ/cm^2^ UVB irradiation, while the UA pretreatment with concentrations of 10, 20, and 40 *μ*M prior to UVB radiation significantly (*P* < 0.01) increased both enzymatic (SOD, CAT, and GPx) and nonenzymatic (GSH) antioxidant activities compared to the UVB-irradiated group. Among the three concentrations of UA, the 20 *μ*M pretreatment greatly increased the intracellular antioxidant status (Figures 5(b) and 5(c)).

### 3.5. Effect of UA on UVB-Induced DNA Damage

**Figure 6:**
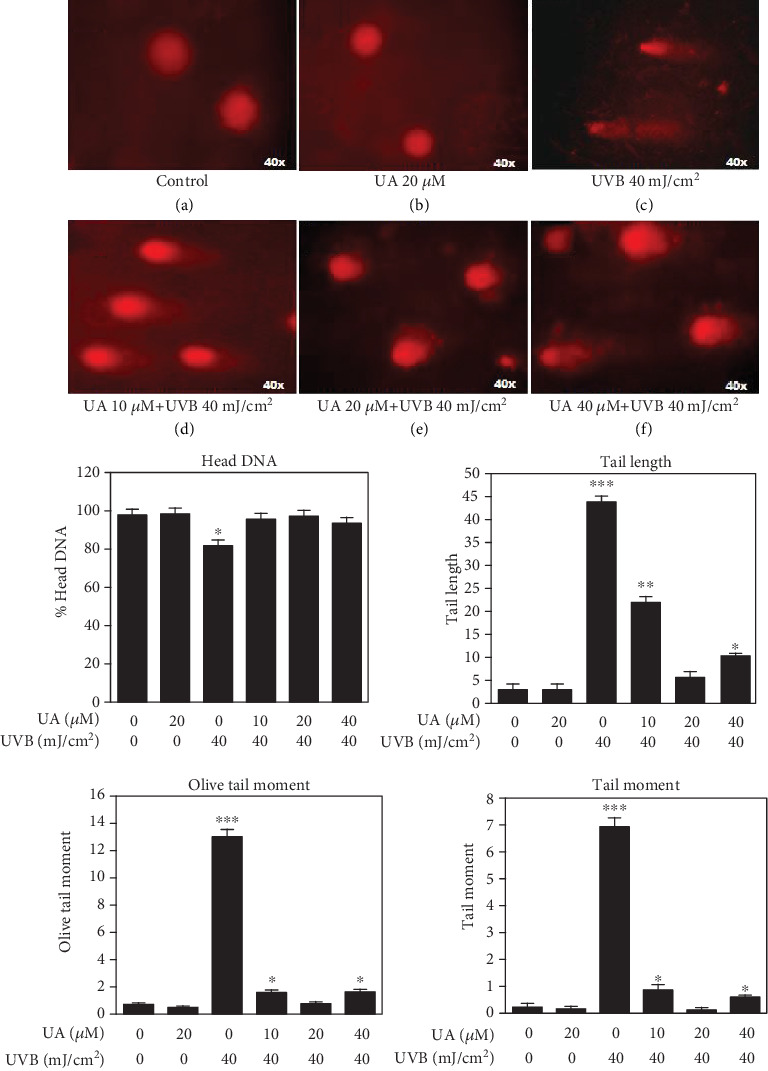
Fluorescent microscopy images of DNA damage in HDFs treated with UA, UVB radiation, and UA+UVB radiation by using comet assay; (a, b) Round nuclei with red staining showing the DNA was not damaged in HDFs treated with UA 20 *μ*M; (c) lack of round nuclei with comet tail length indicated increased DNA damage in HDFs by UVB 40 mJ/cm^2^ irradiation; (d–f) the DNA damage was gradually decreased in HDFs by pretreatment of UA (10, 20, and 40 *μ*M) with UVB 40 mJ/cm^2^ irradiation. Graphs are showing the % head DNA, tail length, tail moment, and Olive tail moment levels. All images were captured at 40x magnification; the bars represent mean ± SD of six separate experiments (*n* = 6 per group). Statistical analysis was performed by one-way ANOVA followed by DMRT at *P* < 0.05, *P* < 0.01, and *P* < 0.001 compared to the nonirradiated control. *P* values not sharing a common marking (∗, ∗∗, and ∗∗∗) differ significantly (DMRT).

The DNA was not affected in control and HDFs treated with UA 20 *μ*M (Figures 6(a) and 6(b)), whereas the DNA was damaged in HDFs treated with UVB 40 mJ/cm^2^ compared to control (Figure 6(c)). The HDFs treated with UA 20 *μ*M+UVB showed a small comet DNA tail length (Figure 6(e)) compared to the comet DNA tail length of the cells treated with UA 10 *μ*M+UVB and UA 40 *μ*M+UVB (Figures 6(d) and 6(f)). Our quantification results showed that UVB-irradiated HDFs significantly increased the levels of DNA tail length, tail moment, and Olive tail moment and decreased % head DNA compared to the control and UA 20 *μ*M treated cells. UA (10, 20, and 40 *μ*M) pretreatment with UVB radiation significant decreased the levels of DNA damage and increased the levels of head DNA in a concentration-dependent manner.

### 3.6. UA Inhibits Nuclear Condensation in UVB-Irradiated HDF Cells

**Figure 7:**
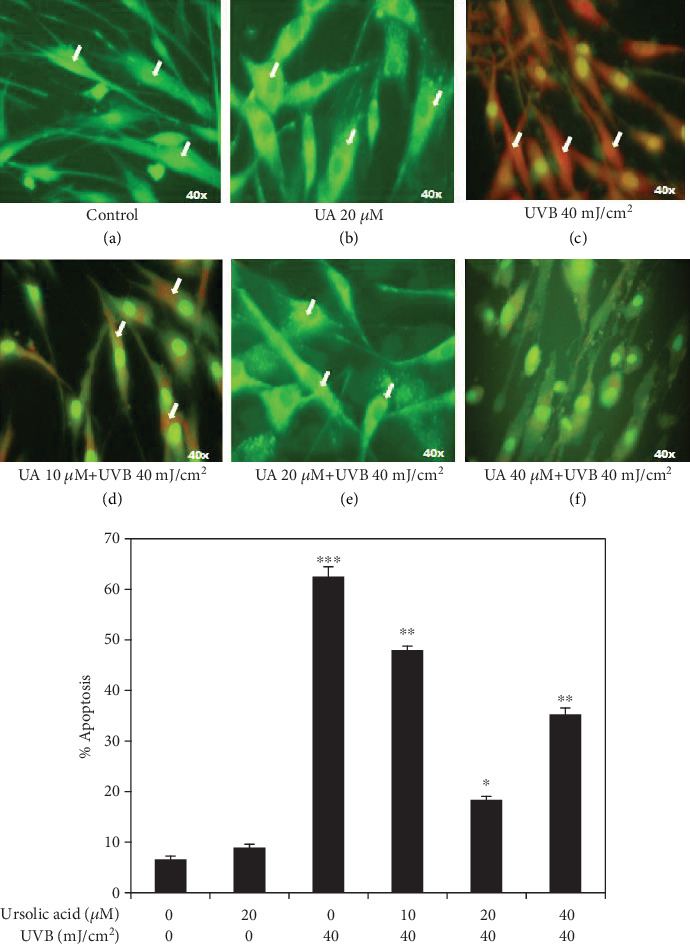
Fluorescent microscopy imaged of acridine orange/ethidium bromide staining to observed the apoptotic morphological changes in HDFs treated with UA, UVB radiation, and UA+UVB radiation; (a, b) green fluorescence of acridine orange accumulation indicating that apoptosis was not induced by UA 20 *μ*M treated cells; (c) red staining of ethidium bromide indicates severe apoptosis induction in HDFs by UVB 40 mJ/cm^2^; (d) red and green fluorescent staining showed moderate apoptosis by UA 10 *μ*M+=UVB 40 mJ/cm^2^; (e, f) strong green staining indicated that apoptosis reduced the treatment of UA 20 *μ*M+UVB 40 mJ/cm^2^ and UA 40 *μ*M+UVB 40 mJ/cm^2^. Data analyses are shown in the graph below images. All images were captured at 40x magnification. Values are given as mean ± SD of six experiments in each group; statistical analysis was performed by one-way ANOVA followed by DMRT at *P* < 0.05, *P* < 0.01, and *P* < 0.001 compared to the nonirradiated control. *P* values not sharing a common marking (∗, ∗∗, and ∗∗∗) differ significantly (DMRT).

The fluorescent DNA-binding dyes including acridine orange and ethidium bromide were used to differentiate cells that are in stages of apoptotic induction and necrotic bodies. Normal healthy cells take up acridine orange (green) whereas ethidium bromide is absorbed by apoptotic cells (red). The HDF cells treated with control and UA 20 *μ*M showed only green color suggesting the absence of apoptosis (Figures 7(a) and 7(b)). Apoptotic morphological changes (red color) significantly increased in the HDF cells treated with UVB irradiation (40 mJ/cm^2^) compared to the control group (Figure 7(c)). The UA 10 *μ*M+UVB and UA 40 *μ*M+UVB induced moderated apoptotic morphological changes (green and red color) in the HDFs (Figures 7(d) and 7(f)) whereas no apoptosis (green color) was observed in HDFs treated with UA 20 *μ*M+UVB (Figure 7(e)).

### 3.7. Effect of UA in Protein Expressions on UVB-Irradiated HDFs

**Figure 8:**
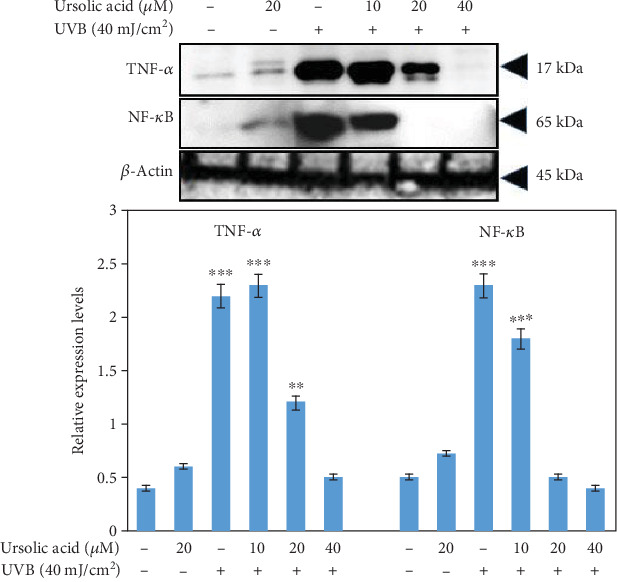
Western blot analysis to detect the expression of TNF-*α* and NF-*κ*B in HDF cells treated with UA, UVB radiation, and UA+UVB radiation. The graph represents the quantification results normalized to *β*-actin levels. Data represent the mean ± SD of three individual experiments. ^∗^Significantly different from control cells (*P* < 0.05). ^∗∗^Significantly different from control cells (*P* < 0.01). ^∗∗∗^Significantly different from control cells (*P* < 0.001).

HDF cells were exposed to UVB (40 mJ/cm^2^) with or without pretreatment of UA (10, 20, and 40 *μ*M). Cells were harvested at 4 h after UVB exposure, and cell lysates were prepared to determine the activation of TNF-*α* and NF-*κ*B using western blot analysis. The expression of TNF-*α* and NF-*κ*B was not affected in control cells and in cells treated with UA 20 *μ*M (Figure 8). The expression of TNF-*α* and NF-*κ*B was severely upregulated when the cells were exposed to UVB 40 mJ/cm^2^ (Figure 8). The expression of TNF-*α* and NF-*κ*B in HDF cells exposed to UVB irradiation was significantly (*P* < 0.01) downregulated by pretreatment with UA at concentrations of 20 and 40 *μ*M, compared to the UVB-irradiated group.

**Figure 9:**
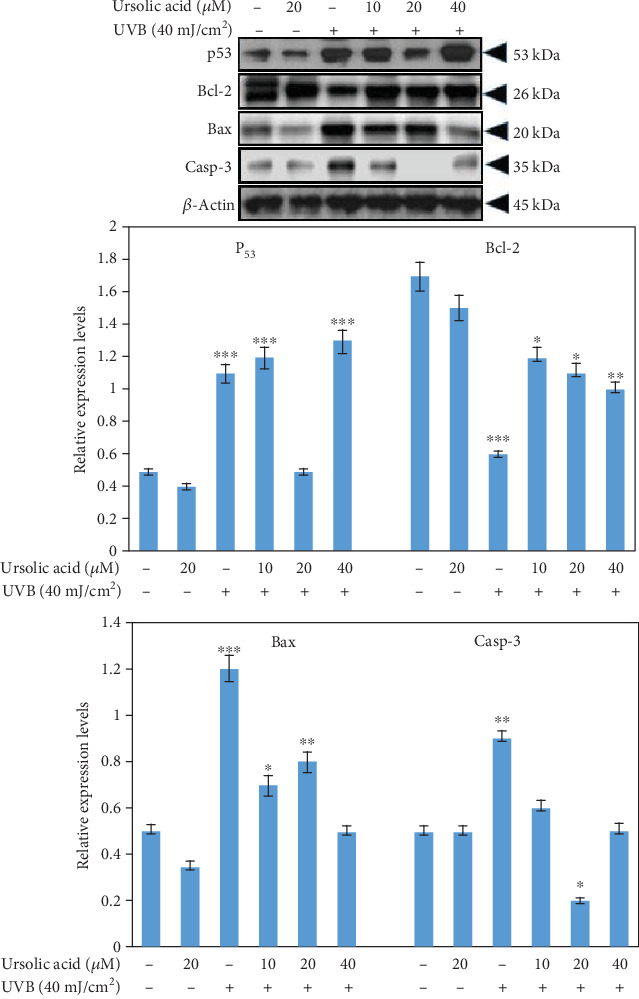
Western blot to detect the expression of p53, Bax, and caspase-3 and Bcl-2 of HDFs treated with UA, UVB radiation, and UA+UVB radiation. The graph represents the quantification results normalized to *β*-actin levels. Data represent the mean ± SD of three individual experiments. ^∗^Significantly different from control cells (*P* < 0.05). ^∗∗^Significantly different from control cells (*P* < 0.01).

Cell lysates were also used to determine the activation of p53, Bax, and caspase-3 and Bcl-2 using western blot analysis. In control and in the cells treated with UA 20 *μ*M, the expression of p53, Bax, and caspase-3 and Bcl-2 was unchanged (Figure 9), whereas the exposure of UVB 40 mJ/cm^2^ significantly upregulated p53, Bax, and caspase-3 and decreased Bcl-2 protein expression (Figure 9). The UA plus UVB (40 mJ/cm^2^) treatment at concentrations of UA 20 and 40 *μ*M significantly downregulated the p53, Bax, and caspase-3 and upregulated Bcl-2 in a dose-dependent manner (compared with the UVB-irradiated group, *P* < 0.05) (Figure 9).

### 3.8. Effect of UA in MMP-2 and 9 Expressions on UVB-Irradiated HDF Cells

**Figure 10:**
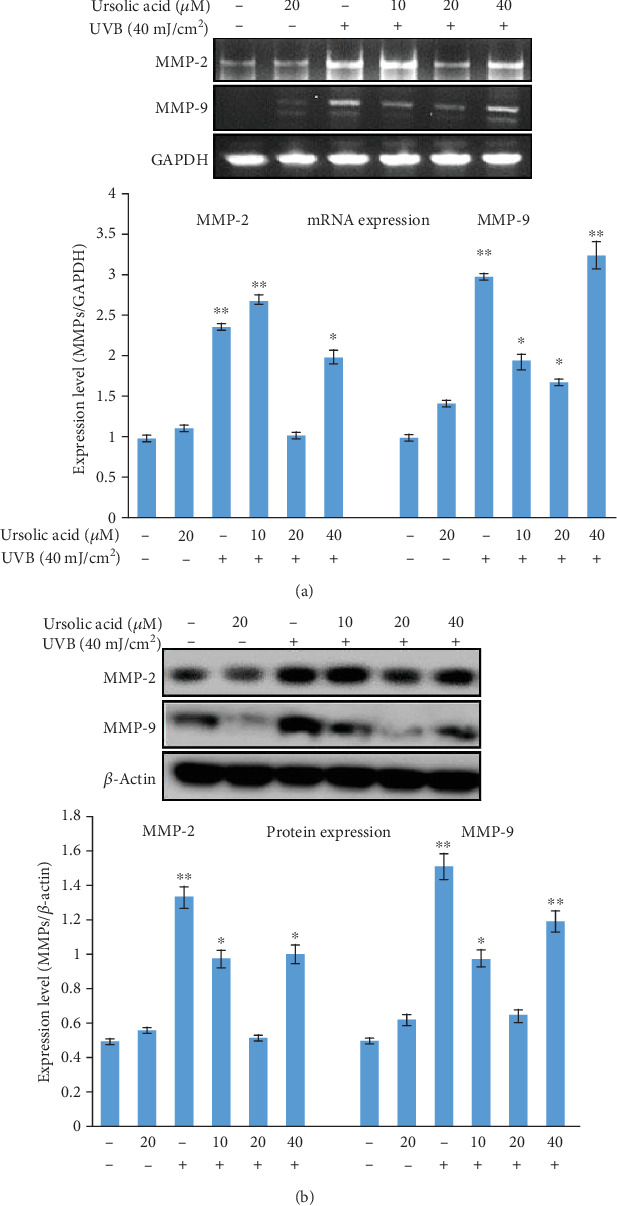
(a, b) Real time-polymerase chain reaction and western blot assays to detect MMP-2 and MMP-9 mRNA and protein expression in HDF cells treated with UA, UVB radiation, and UA+UVB radiation. The graph represents the quantification results normalized to GAPDH and *β*-actin levels. Data represent the mean ± SD of three individual experiments. ^∗^Significantly different from control cells (*P* < 0.05). ^∗∗^Significantly different from control cells (*P* < 0.01).

We examined the antiphotoaging properties of UA on the alteration of MMP-2 and MMP-9 induced by UVB irradiation (Figures 10(a) and 10(b)). The inhibitory role of UA on MMP-2 and MMP-9 mRNA and protein expression induced by UVB irradiation was assessed by PCR and western blot assays. Cells were harvested at 4 h after UVB exposure, and cell supernatants were extracted to determine the activation of MMP-2 and MMP-9 using reverse transcription-PCR and western blot. The MMP-2 and MMP-9 activation was not affected in control cells and in the cells treated with 20 *μ*M UA (Figures 10(a) and 10(b)). UVB (40 mJ/cm^2^) exposure increased the activation of both MMPs in HDFs, while UA pretreatment at concentrations of 20 and 40 *μ*M significantly downregulated the intracellular MMP-2 and MMP-9 activation in the cells exposed to UVB (compared with the UVB-irradiated group, *P* < 0.05).

## 4. Discussion

The skin is a major tissue in the body that presents a flexible and self-repairing barrier and provides protection against a wide range of physiological factors in the environment. Solar ultraviolet radiation, particularly UVB radiation, has amplified by depletion of the stratospheric ozone layer and is known to cause multitiered cellular and molecular abuses eventually leading to both melanoma and nonmelanoma skin cancer [35]. Fibroblasts, which play important roles in the upper dermis, have established extensive protective measures to regulate cellular injury and matrix metalloproteinase activity in response to UVB irradiation [36]. There is considerable evidence that the UVB irradiation induces cellular damage through oxidative stress, peroxidation of DNA, proteins, lipids, and inflammation, factors that have been associated with skin mutagenesis and photocarcinogenesis [37]. Further, the initiation of extracellular matrix damage from UVB-exposed skin could be a result of the activation of the proinflammatory cascades via ROS accumulation leading to cytotoxicity and apoptotic cell death [38]. Our study was conducted to investigate the hypothesis that UA has anti-inflammatory and photoprotective effects on UVB-irradiated HDF cells. The results from the preliminary UVB dose response experiment indicated that the percentage of viable HDFs decreased upon exposure to UVB irradiation compared to the control group. Consequently, we used 40 mJ/cm^2^ in this study, because at this dose, cell viability remains 50%, making it possible to conduct further studies (Figure 1).

The free radical theory is one of the major mechanisms proposed to elucidate the aging process and has received particular attention in skin aging since the skin is constantly exposed to UVB radiation. Skin cell exposure to UVB radiation leads to release of ROS molecules. These free radicals are the most effective trigger of lipid peroxidation and oxidative DNA damage and primary proinflammatory responses [39]. Correspondingly, in this study, we found a high level of ROS generation and disruption of mitochondrial membrane integrity in UVB-irradiated HDFs (Figures 3 and 4). These ROS induced by oxidative stress can ultimately lead to alteration of mitochondrial membrane potential and antioxidant depletion [40]. UA (20 *μ*M) pretreatment decreased the UVB-induced ROS generation and consequently inhibited the mitochondrial membrane potential alteration in HDF cells. Previous studies reported that natural polyphenols such as quercetin, epicatechin, or green tea polyphenols conferred protection to skin cells from UV-induced oxidation damage through free radical scavenging ability [41]. Our previous results also indicated that UA has a photoprotective property by scavenging free radicals in UVB-exposed human blood lymphocytes [42].

In this study, the UVB irradiation-induced lipid peroxidation and antioxidant depletion were used as markers of oxidative damage, and these were significantly inhibited by the treatment with UA (Figures 5(a)–5(c)). Elevated levels of lipid peroxidation have been linked to injurious effects such as loss of fluidity, inactivation of membrane enzymes, increases in permeability to ions, and eventually disruption of cell membrane rigidity leading to damage to cell organelles. Antioxidant enzymes function cooperatively, and a change in any one of them may affect the equilibrium state of oxidative stress [43]. Antioxidant enzymes including CAT, SOD, and GPx protect cellular components from damage by ROS, which represents the primary line of defense mechanism [1]. Our previous study had demonstrated that UVB radiation strongly reduced the activity of these antioxidative enzymes and increased lipid peroxidation products *in vitro* [32]. We observed that UVB irradiation significantly increased the levels of TBARS and decreased the activities of SOD, CAT, GPx, and GSH level; these decreased levels were probably due to irreversible oxidation of the enzyme during neutralization of ROS and lipid peroxidation products. Studies have shown that natural medicinal phytochemicals scavenge ROS to exert a photoprotective effect [6]. Our study has shown that UA pretreatment decreased the generation of ROS to levels low enough to enable the intracellular antioxidant defenses to scavenge the free radicals. An earlier report has shown that UA possesses strong antioxidant and free radical scavenging ability in response to UVB irradiation-induced photosensitization [42]. Thus, UA is a free radical inhibitor, as well as a primary antioxidant that reacts with free radicals. UA may limit the occurrence of free radical damage in normal human cells and promote reversal of lipid peroxidation and antioxidant depletion in UVB-treated HDFs.

Further, UVB radiation is directly absorbed by cellular DNA bases of the skin layer leading to the formation of DNA lesions, mainly cyclobutane pyrimidine dimers (CPDs) and pyrimidine-pyrimidone (6-4) photoproducts, resulting in mutagenesis [1]. Moreover, UVB irradiation has been shown to cause oxidation of guanine residues resulting in the formation of 8-oxo-7,8-dihydro-2-deoxyguanosine (8-oxodG) products from a process mediated by reactive oxygen species (ROS) [42]. UVB-irradiated cells respond to DNA damage by activation of surveillance mechanisms that leads to cell cycle arrest and DNA damage to increase genomic instability. The cellular responses to DNA damage by UVB irradiation are regulated by multiple signaling pathways that initiate DNA damage, cell cycle arrest, and apoptosis [44]. In this study, we observed increasing levels of DNA damage and apoptotic cells after UVB irradiation of HDFs. Pretreatment of UA inhibited UVB irradiation-induced DNA damage and apoptotic cell death in HDFs. Similarly, *in vitro* treatment of pomegranate fruit extract (PFE) was also effective at protecting human skin fibroblasts from cell death following UV exposure and this was related to reduced activation of the nuclear factor-kappa B (NF-*κ*B), a downregulation of caspase-3, and an increased G0/G1 phase associated with DNA repair [45] Further, treatment of HaCaT cells and mouse skin with delphinidin, an anthocyanidin present in pigmented fruits and vegetables, reduced UVB-mediated DNA damage and apoptotic cell death [46].

The intracellular signaling pathway downstream of TNF-*α* and NF-*κ*B plays key roles in regulating cellular senescence and photoaging [47]. UVB irradiation has been shown to increase activation of TNF-*α* in different cells [48], a proinflammatory cytokine known to be an inducer of MMP activation [9]. UVB radiation causes cellular damage via several mechanisms including an induction of cell cycle arrest for DNA damage, or by inducing apoptosis through an activation of apoptotic proteins or by downregulation of antiapoptotic proteins. In our study, the free radicals generated as a result of UVB-irradiated HDFs caused markedly increased apoptosis, which is consistent with the induction of caspase-3. Interestingly, the presence of the free radical scavenger UA, which almost completely attenuated the oxidative stress generated in UVB-irradiated HDFs, also abolished the collapse in membrane potential and the activation of caspase-3 and consequently, rescued cell death. UA-pretreated HDFs showed a decrease in the UVB-induced TNF-*α* and NF-*κ*B expression, resulting in reduction of the photoaging process as shown in Figure 8. Therefore, the present data suggest that UA alleviates photoaging by inhibiting TNF-*α*-induced MMP activation and further reduces photoaging by suppressing p53 production through caspase-dependent cell signaling cascade activation (Figure 9).

Studies have shown that naturally occurring polyphenols, specifically those present in fruits and vegetables, beverages, several herbs, and plants, with diverse pharmacological activities, are a promising class of phytochemicals that inhibit extracellular matrix degradation and cellular alteration [49]. UA has been shown to increase levels of the ceramide and collagen contents in human skin cells [50, 51]. UVB radiation causes skin wrinkles due to the loss of the collagen component of the dermal matrix, which is enhanced by enzymatic antioxidant degradation through the induction of MMPs [11, 52]. UA pretreatment of HDFs inhibited MMP-2 and MMP-9 elevation, as shown in Figures 10(a) and 10(b), and stabilized the extracellular matrix. Based on our findings in this study, we propose that UA may be valuable for cellular protection from UVB radiation-induced cellular photooxidative damage.

Hence, the present study concludes that if the UVB radiation-induced, ROS-mediated DNA lesions are not repaired, these can initiate intracellular signaling and promote photooxidative damage in the skin. We found that UA pretreatment inhibits UVB irradiation-induced cytotoxicity, intracellular ROS generation, changes in mitochondrial membrane potential, lipid peroxidation, antioxidant depletion, DNA damage, and apoptotic induction. Furthermore, UA prevents UVB irradiation-induced apoptotic cell signaling and MMP alteration in HDFs. Besides its antioxidant properties, UA also protects against UVB radiation-induced photoaging process via MMP-mediated structural and extracellular matrix damage.

## Data Availability

The data used to support the findings of this study are included within the article.

## Abbreviations

UVB: Ultraviolet B
HDF: Human skin dermal fibroblast
UA: Ursolic acid.
